# Epidemiology of *Leptospira* Transmitted by Rodents in Southeast Asia

**DOI:** 10.1371/journal.pntd.0002902

**Published:** 2014-06-05

**Authors:** Jean-François Cosson, Mathieu Picardeau, Mathilde Mielcarek, Caroline Tatard, Yannick Chaval, Yupin Suputtamongkol, Philippe Buchy, Sathaporn Jittapalapong, Vincent Herbreteau, Serge Morand

**Affiliations:** 1 INRA, CBGP, Campus international de Baillarguet, CS 30016, Montferrier-sur-Lez, France; 2 Institut Pasteur, Unité de Biologie des Spirochètes, National Reference Center and WHO Collaborating Center for Leptospirosis, Paris, France; 3 Mahidol University, Faculty of Medecine, Siriraj Hospital, Bangkok, Thailand; 4 Institut Pasteur in Cambodia, Virology Unit, Réseau International des Instituts Pasteur, Phnom Penh, Cambodia; 5 Kasetsart University, Department of Parasitology, Faculty of Veterinary Medicine, Bangkok, Thailand; 6 IRD, ESPACE-DEV (IRD, UM2, UR, UAG), Station SEAS-OI, F-97410 Saint-Pierre, France; 7 Institut des Sciences de l'Evolution, CNRS-IRD-UM2, Université de Montpellier 2, Montpellier, France; 8 CIRAD, UR AGIRs, Montpellier, France; 9 Department of Helminthology, Faculty of Tropical Medicine, Mahidol University, Ratchathevi, Thailand; University of Queensland, Australia

## Abstract

**Background:**

Leptospirosis is the most common bacterial zoonoses and has been identified as an important emerging global public health problem in Southeast Asia. Rodents are important reservoirs for human leptospirosis, but epidemiological data is lacking.

**Methodology/Principal Findings:**

We sampled rodents living in different habitats from seven localities distributed across Southeast Asia (Thailand, Lao PDR and Cambodia), between 2009 to 2010. Human isolates were also obtained from localities close to where rodents were sampled. The prevalence of *Leptospira* infection was assessed by real-time PCR using DNA extracted from rodent kidneys, targeting the *lipL32* gene. Sequencing *rrs* and *secY* genes, and Multi Locus Variable-number Tandem Repeat (VNTR) analyses were performed on DNA extracted from rat kidneys for *Leptospira* isolates molecular typing. Four species were detected in rodents, *L. borgpetersenii* (56% of positive samples), *L. interrogans* (36%), *L. kirschneri* (3%) and *L. weilli* (2%), which were identical to human isolates. Mean prevalence in rodents was approximately 7%, and largely varied across localities and habitats, but not between rodent species. The two most abundant *Leptospira* species displayed different habitat requirements: *L. interrogans* was linked to humid habitats (rice fields and forests) while *L. borgpetersenii* was abundant in both humid and dry habitats (non-floodable lands).

**Conclusion/Significance:**

*L. interrogans* and *L. borgpetersenii* species are widely distributed amongst rodent populations, and strain typing confirmed rodents as reservoirs for human leptospirosis. Differences in habitat requirements for *L. interrogans* and *L. borgpetersenii* supported differential transmission modes. In Southeast Asia, human infection risk is not only restricted to activities taking place in wetlands and rice fields as is commonly accepted, but should also include tasks such as forestry work, as well as the hunting and preparation of rodents for consumption, which deserve more attention in future epidemiological studies.

## Author Summary

Leptospirosis is the most prevalent bacterial zoonosis worldwide. Rodents are believed to be the main reservoirs of *Leptospira*, yet little epidemiological research has been conducted on rodents from Southeast Asia. Previous studies suggest that activities which place humans in microenvironments shared by rodents increase the probability of contracting leptospirosis. We therefore investigated the circulation of leptospiral species and strains in rodent communities and human populations in seven localities scattered throughout Southeast Asia; in Thailand, Lao PDR and Cambodia. Molecular typing assays were used to characterize leptospiral species and strains in both rodents and humans, which demonstrated common strains between humans and rodents. Additionally, we observed that the two most abundant leptospiral species; *L. borgpetersenii* and *L. interrogans*, have different habitat requirements, which supposes different modes of transmission. Lastly, in Southeast Asia, the risk of leptospiral transmission to humans is not solely limited to wetlands and rice paddy fields, but is also linked to forested areas, and activities such as the hunting and/or preparation of rodents for consumption.

## Introduction

The World Health Organization (WHO) estimates the global burden of leptospirosis at over one million severe human cases per year, with a growing number of countries reporting leptospirosis outbreaks [1], [2]. Leptospirosis can represent up to 20–40% of idiopathic febrile illness [3], [4]. Symptoms vary widely and mimic those of other diseases, including malaria, viral hepatitis, yellow fever, dengue, bacterial and viral meningitis, as well as many others [5], [6], [7]. Thus, leptospirosis patients may be misdiagnosed with these regionally more common or well-known diseases. In addition, many cases occur in tropical locations without adequate health care, surveillance and reporting, these factors are therefore likely to influence an underestimation of case numbers. For example, Thailand, which has a relatively good health system, reports several thousand cases of leptospirosis each year, while its neighbors, Cambodia and Lao PDR, report very few. This discrepancy is almost certainly due to under-reporting [8], [9].

An additional problem is the limited understanding surrounding basic aspects of the disease epidemiology. Leptospirosis is caused by infection with members of the genus *Leptospira* that includes nine pathogenic species and at least five intermediate species [4]. Most cases of human leptospirosis, however, are not identified at the species, serogroup, or serovar level, hindering environmental risk awareness. In addition to studies aimed at understanding leptospirosis from a genetic standpoint, there have been numerous attempts to understand its transmission. Exposure to virulent leptospires may be direct, via contact with urine or tissues from infected animals, or indirect, where freshwater or humid environments are contaminated with an infected animal's urine. Socio-economic variables and occupations such as mining, cleaning sewers, working in a slaughterhouse, farming, and cattle breeding are known to increase the risk of contracting leptospirosis in Southeast Asia [9], [10], [11].

Limited research has been conducted on the distribution of leptospires in both the environment and in reservoir species. Many species can act as reservoirs, but wild rodents are usually considered to be the main reservoirs for human leptospirosis. Rodents generally acquire leptospirosis as pups, and maintain it as a chronic infection in the renal tubules, excreting bacteria in their urine throughout their life span, often in increasing amounts [6]. Once leptospires are shed into the environment, they can survive in water or soil, depending on physiochemical conditions [10], [12]. In a previous study, Ganoza et al. [13] showed that the concentration and species of leptospires found in environmental surface water correlated with the risk of severe leptospirosis in humans. However, whether leptospiral species have different natural habitat and landscape distribution requirements remains largely unexplored.

In the present study, we aimed to (1) describe *Leptospira* prevalence, species and strains in rodents from seven localities in Southeast Asia (Thailand, Laos PDR and Cambodia); (2) compare isolates from humans living in regions where rodents were sampled; (3) determine whether certain habitats or rodent species increase the prevalence of infection with specific *Leptospira* species. Finally, we discuss the outcomes from this combination of approaches, and their implication for infection routes and environmental risks for humans.

## Methods

### Ethics statement

*Leptospira* cultures from human patients analyzed in this study were previously isolated by the Mahidol University in Thailand as part of the national surveillance for leptospirosis. The strains and DNA samples derived from these cultures were analyzed anonymously for this research study. Systematic field sampling was carried out by joint Asian and French research institute teams. Traps were set within houses with the approval of the owner or tenant. Outdoors, traps were set with the agreement of the village chief. None of the rodent species investigated are on the CITES list, nor the Red List (IUCN). Animals were treated in accordance with the guidelines of the American Society of Mammalogists, and with the European Union legislation (Directive 86/609/EEC). Each trapping campaign was validated by the national, regional and local health authorities. Approval notices for trapping and investigation of rodents were given by the Ministry of Health Council of Medical Sciences, National Ethics Committee for Health Research (NHCHR) Lao PDR, number 51/NECHR, and by the Ethical Committee of Mahidol University, Bangkok, Thailand, number 0517.1116/661. Cambodia has no ethics committee overseeing animal experimentation. The ANR-SEST (*Agence Nationale pour la Recherche, Santé-Environnement et Santé-Travail*) program on rodent-born diseases in Southeast Asia, which provided part of the funding for this project, has been approved by the Managing Directors from both the Asian and French research institutes. In addition, regional approval was obtained from the regional Head of Veterinary Service (Hérault, France), for the sampling and killing of rodents and the harvesting of their tissues (approval no. B 34-169-1) carried out during this study.

### Study sites and rodent trapping

Seven localities were sampled for rodents during 2009 and 2010: Nan (19.15 N; 100.83 E), Loei (17.39 N; 101.77 E) and Buriram (14.89 N; 103.01 E) in Thailand, Luang Prabang (19.62 N; 102.05 E) and Champasak (15.12 N; 105.80 E) in Laos PDR, and Preah Sihanouk (10.71 N; 103.86 E) and Mondolkiri (12.04 N; 106.68 E) in Cambodia.

Within localities, samplings were conducted over an area of about 10 kilometers squared. Four main habitats were distinguished, namely 1) forested (rubber and teak plantations, secondary and primary forest); 2) non-floodable lands (shrubby wasteland, young plantations, orchards), (3) floodable lands (cultivated floodplains, rice fields), and (4) human dwellings (in villages or cities). For each habitat, 10 trapping lines, which each consisted of 10 wire live-traps (hand-made locally, about 40×12×12 cm) every five meters, were installed over a period of four days. Additional captures were also conducted by locals. Captured rodents were collected each day and taken back to the laboratory for dissection according to the protocol of Herbreteau et al. [14].

Where possible, rodent species were determined in the field using morphological criteria from Pages et al. [15], but as morphological criteria were not fully discriminant between some genera, molecular approaches were also carried out. The *mt* gene was used for barcoding *Mus* species and some *Rattus* species (*R. tanezumi, R argentiventer, R. sakeratensis, R. adamanensis*) [16]. In accordance with Pages et al [17], the *mt* lineages “*Rattus* lineage II” and “*Rattus* lineage IV” of Aplin et al [18], were considered as conspecifics and named *R. tanezumi*. Barcoded samples were identified using the webservice RodentSEA [19].

### Leptospira species, strains and genetic diversity

#### PCR detection of *Leptospira*

Real-time polymerase chain reaction (RT-PCR) targeting *lipL32* and *βactin* genes were performed on rodent kidney DNA, extracted with Bio Basic EZ-10 96 Well Plate genomic DNA isolation kit for animals and the Qiagen DNeasy Blood & Tissue Kit. Following alignment of *βactin* gene sequences of Asian rodents [20], the *βactin* primers (forward: 5′-CCA TGA AAC TAC ATT CAA TTC CA-3′; reverse: 5′-TTC TGC ATC CTG TCA GCA A-3′) and probe (5′-AGA CCT CTA TGC CAA CAC AGT GCT G-3′) were designed using the highly conserved fifth exon sequence along with the Probe Design software for Light Cycler LC480 (Roche). *βactin* was used as an internal control for the RT-PCR. The Taqman *lipL32* assay, which targets a gene encoding for a pathogen-specific outer membrane protein in *Leptospira* [21], was performed as previously described by Stoddard et al. [22]. The amplification was performed on a Light Cycler 480 thermocycler (Roche). A Ct<40 for the *lipL32* amplicons was considered positive for *Leptospira*.

#### Genetic characterization of *Leptospira* from rodents

Conventional PCR assays targeting the *rrs* gene were performed on positive RT-PCR samples (Text S1). We first used primers A and B [23] and, if the PCR was negative, we performed a second round of amplification with the inner primers C and RS4. All samples were also amplified for *secY* as previously described [24]. The *rrs* and *secY* PCR products were sequenced at the Platform Genotyping of Pathogens and Public Health (Institut Pasteur, Paris, France).

Multi Locus Variable-number tandem repeat (VNTR) analysis (MLVA) is an alternative DNA amplification-based typing method. This method identifies the number of tandem repeats at VNTR loci throughout the genome by amplifying a given locus with primers targeting flanking regions and then determining the size of the amplified fragment by gel electrophoresis. For *L. interrogans* only, we amplified VNTR-4, VNTR-7, and VNTR-10 loci as previously described by Salaun et al. [25]. In the absence of PCR products, a second round of nested PCR amplification was performed with primers NP4A (5′-TTGGAGCGCAATCTCTTTTT-3′) and NP4B (5′- TGAGGATACCCCATTTTTACCTT-3′), NP7A (5′-GATGGGCGGAGAAAAGTGTA-3′) and NP7B (5′-TGGATCGGTATTTTGGTTCA- 3′), NP10A (5′- ATTCCAAAACTCAGCCCTCA-3′) and NP10B (5′- TGATGGGATTACCGGAAGAA-3′).

#### Human samples

In order to compare the genetic typing of rodent *Leptospira* DNA with *Leptospira* strains obtained from humans, we analyzed DNA from 15 human isolates, which were maintained by the Mahidol University, Siriraj Hospital, Faculty of Medicine, Department of Medicine (Text S1). These samples were collected in close proximity to the rodent-sampling locations.

#### *Leptospira* species and strain identification

*Leptospira* species were identified by sequencing *rrs* genes and subsequent BLAST searches using the GenBank database (http://www.ncbi.nlm.nih.gov/BLAST). Strain characterization below the species level, was performed using both *secY* gene [26], [27], [28], [29] and MLVA analyses [25]. *secY* genotypes were compared to the MLST database of Nalam et al. [29], which contains data from 271 isolates from different geographical areas, as well as sequences found in the NCBI database. MLVA patterns were compared to the National Reference Center (NRC) database for Leptospirosis (Institut Pasteur, Paris, France), which contains more than one hundred genotypes of *Leptospira* isolates.

Phylogenetic analyses of *secY* were performed with the Neighbour-Joining method using Kimura 2-parameters distance with Seaview software v.4.2.12 [30] and visualized in FigTree v1.3.1 (http://tree.bio.ed.ac.uk/). Nodal supports were determined using the bootstrap approach (1000 replicates).

#### Statistical analyses

We investigated the effects of host species, sex, maturity (young *vs* adult) and environmentally-linked variables (habitat and locality) on the infection status of individual rodents. Statistical logistic regressions were performed with the R statistical platform using the package MuMIn v.1.7.2 [31] and lme4 [32]. Model selection was performed using Akaike's Information Criterion (AIC) [33], [34]. The model with the lowest AIC value was viewed as the most parsimonious, i.e. the model which explains the majority of variance with the fewest parameters [33]. The significance of each explanatory variable was tested using Wald tests based on z values.

## Results

### *Leptospira* prevalence, species and strains

**Figure 1:**
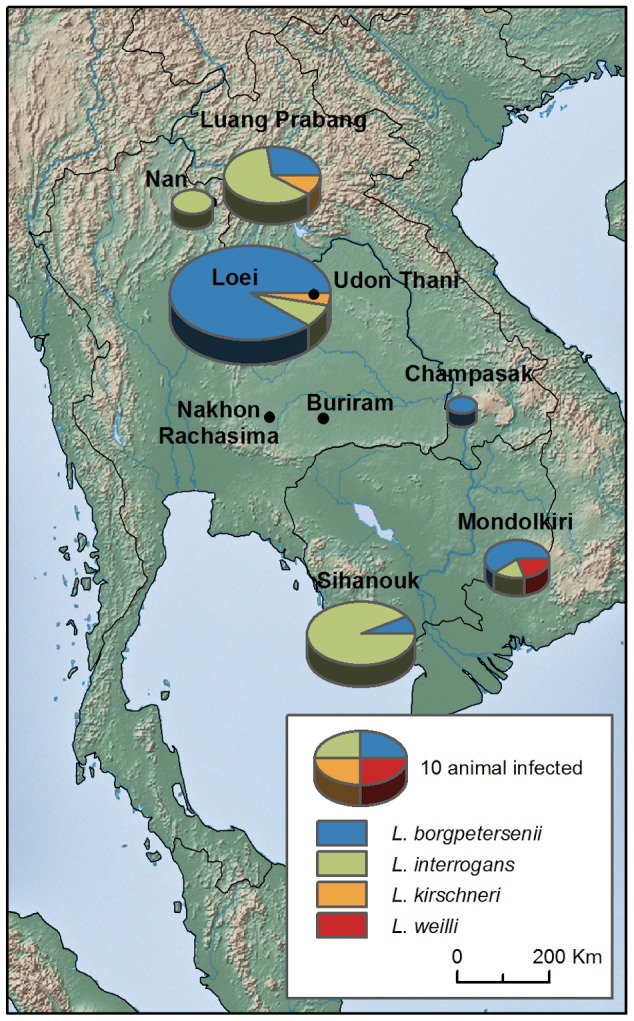
Geographic distribution of *Leptospira* infection in rodents from Thailand, Lao PDR and Cambodia.

We detected 64 *Leptospira*-positive rodents from the 901 tested, giving a mean prevalence of 7.1%. Nineteen shrews (*Suncus murinus*) were also tested and all were found negative. Leptospires were detected in six localities (Figure 1) with highly variable prevalence across localities, from 0% to 18%. Twelve rodent species (over 18 tested) were found positive and prevalence varied from 0 to 19% across species (Table 1).

**Table 1:** Prevalence of *Leptospira* species according to locality and rodent species from Thailand, Lao PDR and Cambodia.

Country	Locality	Nb samples	Prevalence (%)	*borgpetersenii*	*interrogans*	*kirschneri*	*weilii*
Thailand	Loei	174	17.81	28	2	1	0
	Nan	94	2.12	0	2	0	0
	Buriram	129	0.00	0	0	0	0
Lao PDR	Luang Prabang	140	7.85	3	7	1	0
	Champasak	71	1.41	1	0	0	0
Cambodia	Sihanouk	168	8.33	1	13	0	0
	Mondolkiri	125	4.00	3	1	0	1
**Species**							
*Bandicota*	*indica*	27	3.70	0	1	0	0
*Bandicota*	*savilei*	52	1.92	0	0	0	1
*Berylmys*	*berdmorei*	13	15.38	1	1	0	0
*Berylmys*	*bowersi*	1	0.00	0	0	0	0
*Leopoldamys*	*edwardsi*	3	0.00	0	0	0	0
*Maxomys*	*surifer*	43	6.98	1	2	0	0
*Mus*	*caroli*	88	5.68	5	0	0	0
*Mus*	*cervicolor*	65	9.23	5	0	1	0
*Mus*	*cookii*	85	18.82	14	1	1	0
*Niviventer*	*fulvescens*	14	0.00	0	0	0	0
*Rattus*	*andamanensis*	4	0.00	0	0	0	0
*Rattus*	*argentiventer*	37	13.51	1	4	0	0
*Rattus*	*exulans*	220	0.45	0	1	0	0
*Rattus*	*losea*	47	12.77	5	1	0	0
*Rattus*	*nitidus*	6	0.00	0	0	0	0
*Rattus*	*norvegicus*	10	0.00	0	0	0	0
*Rattus*	*tanezumi*	186	9.68	4	14	0	0
Total		901	7.10	36	25	2	1

The *rrs* PCR assay was performed on the 64 samples which were positive for *Leptospira*. After 25 of these samples returned negative results following direct PCR, they were then analyzed by nested PCR. All PCR products were then sequenced and the *Leptospira* species were categorized based on phylogenetic analysis of the *rrs* fragment. Four species were determined: *L. borgpetersenii* (n = 36), *L. interrogans* (n = 25), *L. kirschneri* (n = 2) and *L. weilli* (n = 1).

**Figure 2:**
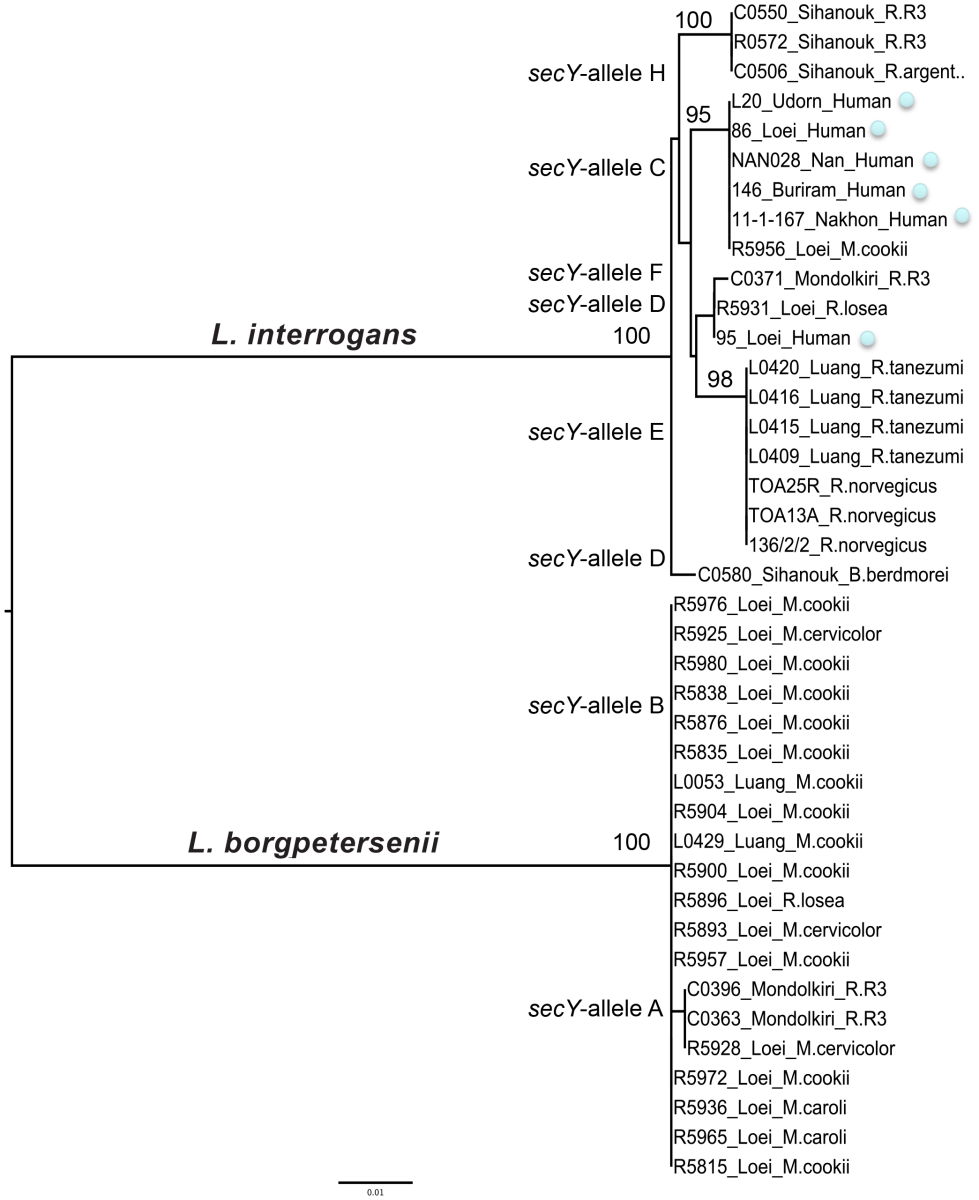
Phylogenetic analysis for the *secY* gene of *Leptospira sp.* isolated from rodents and humans from Thailand, Lao PDR and Cambodia. Information about locality, rodent species and/or human cases are indicated. See Text S1 for details about samples. Numbers above branches are bootstrap values (only >0.90 are indicated).

The amplification of *secY* was successful in 31 of the 64 samples positive for *Leptospira*, including 20 *L. borgpetersenii* and 11 *L. interrogans*. No amplification could be detected for *L. kirschneri* and *L. weilli*. Lack of amplification was probably due to low levels of *Leptospira* DNA in the samples. The alignment of the 549-bp *secY* fragments distinguished a total of eight distinct alleles (GenBank accession numbers: KF770694-KF770731), including two alleles for *L. borgpetersenii* (A and B) and six for *L. interrogans* (C to H). There was no clear association between *secY*-identified strains and either locality or rodent species (Figure 2 and Text S1). MLVA positively identified 15 of the 25 tested samples, which were positive for *L. interrogans* (Text S1). The MLVA patterns are in close agreement with the alleles determined by *secY* sequencing. Comparison with our reference strains indicates that our samples share an identical *secY* sequence and MLVA profile to strains of the Canicola, Pyrogenes and Autumnalis serogroups (Text S1).

### Correspondence with strains and serovars isolated from humans

Two *secY* alleles from *L. interrogans* were recovered from both rodents and humans (Figure 2, Text S1). The *secY* C allele was recovered from the wild mouse, *M. cookie*, from Loei, northern Thailand. This allele had previously been characterized from the ST34 clone, which corresponds to a Autumnalis serogroup human isolate associated with the northern Thailand outbreak between 1999 and 2003 (Thaipadungpanit et al. 2007). Secondly, the *secY* D allele (MLVA pattern 640/750/650) of the Pyrogenes serogroup was found in both rodent and human samples from Loei, northern Thailand.

### Determinants of rodent infection

**Figure 3:**
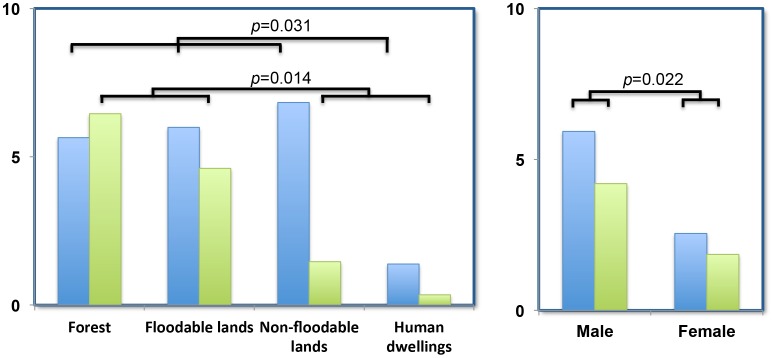
Prevalence of *L. borgpetersenii* (blue) and *L. interrogans* (green) in rodents according to habitat and sex. *p*-values are indicated.

Statistical analysis revealed that rodent locality, habitat and sex, significantly affected individual infection (Table 2). Rodents living in households showed significantly lower infection rates (Figure 3). Males were significantly more likely to be infected than females (Figure 3). By contrast, rodent species showed no correlation with infection. As there was potential non-independence between the distributions of rodent species among habitats; as three species are strictly restricted to households; we re-analyzed the data after removing all household rodent data. We found similar results (data not shown), again with significant effects due to locality and sex, but not species, indicating that those species living outside human dwellings have an overall similar level of infection.

**Table 2:** General Linear Model of rodent infection by *Leptospira* with binomial distribution and logit link function (Log-Likelihood Type 1 Test).

Variables	Estimates (SD)	*p*-values	Deviance	Degree freedom	AIC
Sex: Male	0.66 (0.29)	0.022*			
Habitat: Floodable lands	0.24 (0.40)	0.551			
Habitat: NFLoodable lands	−0.44 (0.38)	0.249			
Habitat: Human dwellings	−1.63 (0.54)	0.002**			
Locality: Buriram	−3.51 (1.03)	<0.001***			
Locality: Champasak	−2.31 (1.04)	0.026*			
Locality: Luang Prabang	−0.79 (0.40)	0.048*			
Locality: Mondolkiri	−1.51 (0.52)	0.003**			
Locality: Nan	−1.86 (0.76)	0.014*			
Locality: Sihanouk	−0.47 (0.37)	0.202			
Intercept	−1.61 (0.41)	<0.001***			
			452.2	837	401.9

* significant;

** very significant;

*** highly significant.

Selection of the best model using AIC criterion, with initial model with locality, habitat, species, sex and maturity as explicative variables.

*L. borgpetersenii* and *L. interrogans* infection were then investigated separately, which once again showed that locality, habitat and sex, but not rodent species, were the major determinants of infection (Figure 3 and Text S1). This last analysis suggested a difference in ecological niche for both *Leptospira* species. In particular, *L. borgpetersenii* was much more abundant in dry habitats (non-floodable lands) than *L. interrogans*.

## Discussion

Using a combination of molecular and ecological data, we have been able to document the circulation of *Leptospira spp.* within Southeast Asian rodent communities. We were able to determine the genotypes of isolates infecting rodents, by direct amplification of kidney DNA, without the need to culture the pathogen. Our data support the premise that rodent infection is mainly driven by habitat rather than rodent species. Moreover, the two most abundant *Leptospira* species have contrasting habitat requirements: *L. interrogans* is restricted to humid habitats (rice fields, forests and floodable lands) while *L. borgpetersenii* was equally abundant in floodable lands, forests and non-floodable lands. Finally, two leptospiral strains were found in common between humans and rodents, consistent with the theory that rodents transmit the disease to humans [35]. Therefore, human infection risk may not just be limited to wetlands, as currently believed. The circulation of *Leptospira* species is also high in forested and non-floodable lands, suggesting that human activities like forest work, hunting and preparing rodents for their consumption, deserve more attention in future epidemiological studies.

### Ecological drivers of *Leptospira* infection in rodents

Most research on the presence of leptospires in rodents has been conducted in urban areas, or rural areas in the vicinity of households [36], [37], [38]. In contrast, only a few studies have investigated the prevalence of *Leptospira* in rodents within their various habitats [39]. Our study suggested that the mean prevalence in rodents across localities was approximately 10%, when we excluded rodents trapped in human dwellings where prevalence was very low (2%). *Leptospira* prevalence was similar between floodable areas, forests and non-floodable agricultural fields. Our results then challenge the widely accepted belief that leptospires mainly circulate in wetlands. Two potential leptospirosis transmission routes are generally assumed; direct transmission between individuals, or via the external environment. The relative importance of these routes in rodents is unknown; however, our results suggest that direct transmission could explain the circulation of leptospires in dry habitats.

Individual variation in susceptibility to infection is a common outcome of epidemiological studies. In the context of pathogenesis, infection may vary with numerous individual features such as sex, age, physiological condition, behavior and immunogenetics [40], [41]. Recently, Perez et al showed that meteorological conditions might also influence *Leptospira* carriage in rodents, with hot and rainy seasons associated with both high abundance and increased prevalence in rodents [38]. Taking into account this inter-individual variability greatly enhances both our understanding of disease epidemiology, and our ability to predict the outcomes of epidemics by using adapted epidemiological models [42]. Statistical analyses of our dataset revealed that males were clearly more susceptible to *Leptospira* infections than females, consistent with many reports on vertebrate infections [43]. Differing infection rates observed due to sex might result from endocrine-immune interactions. Androgens have immunosuppressive effects, explaining the reduced efficiency of the male immune system and its association with higher infection rates. Moreover, steroid hormones alter rodent behavior which then influences susceptibility to infection. Males of most mammals are more aggressive than females, more likely to disperse, and have larger home ranges with more intense foraging activities; all these behaviors cause increased pathogen exposure.

Additionally, rodent susceptibility to *Leptospira* infection did not significantly vary across rodent species. Most rodent species were found to be infected by *Leptospira* and our statistical modelling did not highlight “species” as a significant factor explaining *Leptospira* infection. The observed variation in prevalence across rodent species is most probably an indirect consequence of their specific habitat requirements. For instance the Pacific rat, *Rattus exulans*, was rarely found infected, but this probably results from its close association with human dwellings where *Leptospira* prevalence is consistently lower than in other habitats. Although rarely documented, the different rodent species investigated here display clear habitat preferences [39], [44]. Some species are more abundant in rain-fed paddy fields (*Bandicota indica, R. argentiventer*) or forests (*Leopoldamys edwardsi, Maxomys surifer*) or non-flooded fields (*Mus cervicolor, Mus cooki*). In line with other studies [39], [45], our results confirmed the importance of *Bandicota* and *Rattus* species as hosts for *Leptospira* strains of human health importance. However, high prevalence of pathogenic species and strains were also observed in rarely investigated rodents such as forest species (*Berylmys sp.*, *Maxomys sp.*) and wild mice (*Mus sp.*). This observation suggests that rodent reservoirs for human leptospirosis are probably more diverse than previously thought (see [35]).

Finally we discuss any of the present study's limitations, which may mitigate some of the above interpretations. As reported in other agricultural systems (see [46], [47] for instance) rodents may move among habitats, either as part of the dispersal process (i.e. the movement of an organism from its birth place to its first breeding site, or from one breeding site to another), or in response to the seasonal variation in habitat quality (i.e. amount of food, shelter availability, competition with other rodents, predation etc.). In the case of Southeast Asian rodents, one can imagine seasonal movements between flooded and non-flooded habitats, or between other habitats, but we lack data on these movements, which have not been the subject of publication to our knowledge. Because these movements may involve rodents infected with *Leptospira*, this process could have important consequences on *Leptospira* distributions within Southeast Asian agricultural landscapes (see [48] for an example of the importance of rodent movements for the epidemiology of a rodent-borne hantavirus in Europe). As has already been pointed out by Singleton and collaborators [49], more data is needed on the ecology of rodents in Southeast Asia, and such data would probably significantly increase our understanding of *Leptospira* epidemiology.

### Contrasting transmission routes for *L. interrogans* and *L. borgpetersenii*

Another insight of our study is that the two most abundant *Leptospira* species, *L. interrogans* and *L. borgpetersenii*, both of which are of great significance to human disease in Asia [35], [50], [51], [52], may have different epidemiological cycles. *L. interrogans* infection in rodents was restricted to humid habitats while *L. borgpetersenii* infection was equally frequent in both humid and dry habitats. This new ecological data on rodents is consistent with previous data gained from experimental and genomic studies. Experimental data suggest that survival in water is highly reduced for some strains of *L. borgpetersenii* when compared to *L. interrogans*. *L. borgpetersenii* serovar Hardjo lost >90% viability after 48 h in water, whereas *L. interrogans* retained 100% viability over the same period [53]. *L. interrogans* would thus be able to survive in such an environment, especially in surface water, allowing transmission from contaminated water. Whereas *L. borgpetersenii* would not survive outside its host, forcing direct host-to-host transmission. This difference in ecological niche is reflected in the genomic composition of the two species. *L. borgpetersenii* serovar Hardjo strains have a smaller genome than *L. interrogans*. Genome rearrangement in these strains of *L. borgpetersenii* mainly affect the ability to sense the external environment, which may indicate that these strains are in the process of becoming specialized for direct transmission. In contrast, *L. interrogans* has many environmental sensing genes and exhibits large shifts in protein expression when moved from a natural environment-like medium to a host-like medium [54]. While we cannot presume that the change reported by Bulach et al. [53] is representative of all *L. borgpetersenii* strains or is only restricted to certain strains, their study demonstrates that genome composition and habitat preference may largely differ across strains and species. These *in vitro* results are consistent with our ecological observations.

Whether environmental conditions (outside the host) determine *Leptospira* species distribution in nature remains largely unexplored. Ganoza et al. [13] showed differential distribution of isolates in urban or rural water sources in Peru, reflecting rates found among human isolates from both urban and rural settings. Perez et al [38] demonstrated that seasonal variations influence *Leptospira* prevalence in rats and mice from New Caledonia. Very little data has been published concerning the epidemiology of both *Leptospira* in humans and wildlife, however some human epidemiology reports suggest that *L. interrogans* is commonly acquired from contaminated surface water, whereas a host-to-host transmission cycle is more likely to occur for *L. borgpetersenii* [53]. However our results suggest a lower transmission risk from rodents to humans for local *L. borgpetersenii* strains, in comparison with *L. interrogans* strains.

### Conclusion

Most studies in Southeast Asia currently focus on human infection linked to humid habitats and rice cultivation [9], [49]. Without calling into question the importance of this route of transmission, our results suggest alternate routes of infection, which deserves further study. Human infection could also occur in other humid habitats, such as standing water and forest streams. Moreover, rodents are the subject of traditional hunting and trade in many parts of Southeast Asia. Close contact between rodents and humans during these activities, as well as rodent preparation before consumption, could present a significant route of infection, which should be evaluated. In line with our results, frequent human activity in forests was identified as a significant risk factor in Laos [9]. On the other hand, and fortunately, human contamination by commensal small mammals is probably low in Southeast Asia, despite the abundance of rodents and shrews in human dwellings. To our knowledge, our work is the first ecological evidence supporting different transmission routes for *L. interrogans* and *L. borgpetersenii* species in nature. Clearly this last point deserves more study, notably in order to strictly demonstrate the predominance of *L. borgpetersenii* direct transmission in ecological systems, as well as to determine if this transmission mode holds true for all *borgpetersenii* serovars, or for only some specific serovars. Together, this work brings to light novel perspectives on leptospiral epidemiology, reinforces the existence of species-specific transmission routes in nature, and stresses the need for the precise diagnosis of *Leptospira* involved in human and animal infections in order to better understand and foresee epidemics.

## Supporting Information

Text S1 **Table S1.** List of leptospires characterized in rodents from seven localities in Thailand, Lao PDR and Cambodia. *Leptospira* species identification relied on *rrs* gene sequencing. We also indicate information about molecular results of the *secY* gene sequencing (strain A to G), MLVA typing and capture data. ND: non-determined, FL: floodable lands; NFL: non-floodable lands, F: forest, HD: human dwellings. **Table S2.** List of reference and clinical strains used in this study. We indicate information about species, serogroup, host, sampling and molecular results of the *secY* gene sequencing (alleles A to G) and MLVA typing. ND = non-determined. **Table S3.** General Linear Model of rodent infection by *Leptospira borgpetersenii* with binomial distribution and logit link function (Log-Likelihood Type 1 Test). Selection of the best model using AIC criterion, with initial model with locality, habitat, species, sex and maturity as explicative variables **Table S4.** General Linear Model of rodent infection by *Leptospira interrogans* with binomial distribution and logit link function (Log- Likelihood Type 1 Test). Selection of the best model using AIC criterion, with initial model with locality, habitat, species, sex and maturity as explicative variables.
(DOCX)
