# Supplementary material for: Epidemiology of Leptospira Transmitted by Rodents in Southeast Asia
Source: PLoS Negl Trop Dis. 2014 Jun 5;8(6):e2902. doi: 10.1371/journal.pntd.0002902 (PMC4046967; doi:10.1371/journal.pntd.0002902)
Supplement: Text S1 — Table S1. List of leptospires characterized in rodents from seven localities in Thailand, Lao PDR and Cambodia. Leptospira species identification relied on rrs gene sequencing. We also indicate information about molecular results of the secY gene sequencing (strain A to G), MLVA typing and capture data. ND: non-determined, FL: floodable lands; NFL: non-floodable lands, F: forest, HD: human dwellings. Table S2. List of reference and clinical strains used in this study. We indicate information about species, serogroup, host, sampling and molecular results of the secY gene sequencing (alleles A to G) and MLVA typing. ND = non-determined. Table S3. General Linear Model of rodent infection by Leptospira borgpetersenii with binomial distribution and logit link function (Log-Likelihood Type 1 Test). Selection of the best model using AIC criterion, with initial model with locality, habitat, species, sex and maturity as explicative variables Table S4. General Linear Model of rodent infection by Leptospira interrogans with binomial distribution and logit link function (Log- Likelihood Type 1 Test). Selection of the best model using AIC criterion, with initial model with locality, habitat, species, sex and maturity as explicative variables. (DOCX) [file pntd.0002902.s001.docx]

**Table S1.** List of leptospires characterized in rodents from seven localities in Thailand, Lao PDR and Cambodia. *Leptospira* species identification relied on *rrs* gene sequencing. We also indicate information about molecular results of the *secY* gene sequencing (strain A to G), MLVA typing and capture data. ND: non-determined, FL: floodable lands; NFL: non-floodable lands, F: forest, HD: human dwellings.

| ***Leptospira* sp.** | ***secY*** | **MLVA ^b^** | **Country** | **Locality** | **Habitat** | **Rodent** | **Sex** | | **ID** |
| --- | --- | --- | --- | --- | --- | --- | --- | --- | --- |
| *L.borgpetersenii* | ND | ND | Lao PDR | Champasak | FL | *Rattus losea* | M | L0270 | |
| *L.borgpetersenii ^a^* | ND | ND | Thailand | Loei | F | *Berylmys berdmorei* | M | R5990 | |
| *L.borgpetersenii ^a^* | ND | ND | Thailand | Loei | NFL | *Maxomys surifer* | M | R5999 | |
| *L.borgpetersenii* | ND | ND | Thailand | Loei | FL | *Mus caroli* | M | R5827 | |
| *L.borgpetersenii ^a^* | ND | ND | Thailand | Loei | FL | *Mus caroli* | M | R5929 | |
| *L.borgpetersenii* | B | ND | Thailand | Loei | FL | *Mus caroli* | M | R5936 | |
| *L.borgpetersenii* | B | ND | Thailand | Loei | NFL | *Mus cookii* | M | R5957 | |
| *L.borgpetersenii* | B | ND | Thailand | Loei | FL | *Mus caroli* | M | R5965 | |
| *L.borgpetersenii ^a^* | ND | ND | Thailand | Loei | FL | *Mus caroli* | F | R5985 | |
| *L.borgpetersenii ^a^* | ND | ND | Thailand | Loei | NFL | *Mus cervicolor* | M | R5816 | |
| *L.borgpetersenii* | B | ND | Thailand | Loei | NFL | *Mus cervicolor* | M | R5893 | |
| *L.borgpetersenii ^a^* | ND | ND | Thailand | Loei | NFL | *Mus cervicolor* | F | R5919 | |
| *L.borgpetersenii* | B | ND | Thailand | Loei | FL | *Mus cervicolor* | M | R5925 | |
| *L.borgpetersenii* | A | ND | Thailand | Loei | NFL | *Mus cervicolor* | M | R5928 | |
| *L.borgpetersenii* | B | ND | Thailand | Loei | F | *Mus cookii* | M | R5815 | |
| *L.borgpetersenii* | ND | ND | Thailand | Loei | NFL | *Mus cookii* | M | R5828 | |
| *L.borgpetersenii* | B | ND | Thailand | Loei | FL | *Mus cookii* | F | R5835 | |
| *L.borgpetersenii* | B | ND | Thailand | Loei | FL | *Mus cookii* | M | R5838 | |
| *L.borgpetersenii* | B | ND | Thailand | Loei | NFL | *Mus cookii* | F | R5876 | |
| *L.borgpetersenii* | B | ND | Thailand | Loei | FL | *Mus cookii* | F | R5900 | |
| *L.borgpetersenii* | B | ND | Thailand | Loei | NFL | *Mus cookii* | M | R5904 | |
| *L.borgpetersenii ^a^* | ND | ND | Thailand | Loei | F | *Mus cookii* | F | R5964 | |
| *L.borgpetersenii* | B | ND | Thailand | Loei | NFL | *Mus cookii* | M | R5972 | |
| *L.borgpetersenii* | B | ND | Thailand | Loei | NFL | *Mus cookii* | F | R5976 | |
| *L.borgpetersenii* | B | ND | Thailand | Loei | NFL | *Mus cookii* | M | R5980 | |
| *L.borgpetersenii* | B | ND | Thailand | Loei | FL | *Rattus losea* | M | R5896 | |
| *L.borgpetersenii ^a^* | ND | ND | Thailand | Loei | FL | *Rattus losea* | F | R5942 | |
| *L.borgpetersenii ^a^* | ND | ND | Thailand | Loei | FL | *Rattus losea* | M | R5944 | |
| *L.borgpetersenii ^a^* | ND | ND | Thailand | Loei | FL | *Rattus losea* | F | R5970 | |
| *L.borgpetersenii ^a^* | B | ND | Lao PDR | Luang Prabang | NFL | *Mus cookii* | M | L0053 | |
| *L.borgpetersenii* | B | ND | Lao PDR | Luang Prabang | NFL | *Mus cookii* | F | L0429 | |
| *L.borgpetersenii* | ND | ND | Lao PDR | Luang Prabang | F | *Rattus tanezumi* | M | L0396 | |
| *L.borgpetersenii* | ND | ND | Cambodia | Mondolkiri | F | *Rattus tanezumi* | F | C0359 | |
| *L.borgpetersenii* | A | ND | Cambodia | Mondolkiri | F | *Rattus tanezumi* | F | C0363 | |
| *L.borgpetersenii* | A | ND | Cambodia | Mondolkiri | F | *Rattus tanezumi* | M | C0396 | |
| *L.borgpetersenii ^a^* | ND | ND | Cambodia | Sihanouk | HD | *Rattus argentiventer* | M | C0642 | |
| *L.interrogans ^a^* | C | ND | Thailand | Loei | NFL | *Mus cookii* | M | R5956 | |
| *L.interrogans* | D | 640/750/650 | Thailand | Loei | FL | *Rattus losea* | M | R5931 | |
| *L.interrogans ^a^* | ND | 300/220/350 | Lao PDR | Luang Prabang | NFL | *Rattus tanezumi* | M | L0083 | |
| *L.interrogans* | ND | 300/220/350 | Lao PDR | Luang Prabang | F | *Rattus tanezumi* | M | L0398 | |
| *L.interrogans* *^a^* | ND | ND | Lao PDR | Luang Prabang | F | *Rattus tanezumi* | M | L0406 | |
| *L.interrogans* | E | 430/350/460 | Lao PDR | Luang Prabang | F | *Rattus tanezumi* | M | L0409 | |
| *L.interrogans* | E | 430/350/460 | Lao PDR | Luang Prabang | F | *Rattus tanezumi* | F | L0415 | |
| *L.interrogans* | E | 430/350/460 | Lao PDR | Luang Prabang | F | *Rattus tanezumi* | F | L0416 | |
| *L.interrogans* | E | 430/350/460 | Lao PDR | Luang Prabang | F | *Rattus tanezumi* | F | L0420 | |
| *L.interrogans* | F | 640/750/650 | Cambodia | Mondolkiri | F | *Rattus tanezumi* | M | C0371 | |
| *L.interrogans* | ND | 430/350/460 | Thailand | Nan | FL | *Bandicota indica* | M | R6072 | |
| *L.interrogans* | ND | 430/350/460 | Thailand | Nan | FL | *Rattus tanezumi* | M | R6096 | |
| *L.interrogans* | G | 620/720/820 | Cambodia | Sihanouk | FL | *Berylmys berdmorei* | M | C0580 | |
| *L.interrogans ^a^* | ND | ND | Cambodia | Sihanouk | HD | *Maxomys surifer* | F | C0513 | |
| *L.interrogans ^a^* | ND | ND | Cambodia | Sihanouk | NFL | *Maxomys surifer* | F | C0584 | |
| *L.interrogans* | H | 430/340/460 | Cambodia | Sihanouk | FL | *Rattus argentiventer* | M | C0506 | |
| *L.interrogans ^a^* | ND | 500/500/460 | Cambodia | Sihanouk | FL | *Rattus argentiventer* | M | C0515 | |
| *L.interrogans ^a^* | ND | ND | Cambodia | Sihanouk | HD | *Rattus argentiventer* | M | C0531 | |
| *L.interrogans ^a^* | ND | ND | Cambodia | Sihanouk | FL | *Rattus argentiventer* | M | C0564 | |
| *L.interrogans ^a^* | ND | ND | Cambodia | Sihanouk | HD | *Rattus exulans* | F | C0676 | |
| *L.interrogans ^a^* | ND | ND | Cambodia | Sihanouk | HD | *Rattus tanezumi* | F | C0507 | |
| *L.interrogans* | H | 430/340/460 | Cambodia | Sihanouk | FL | *Rattus tanezumi* | M | C0550 | |
| *L.interrogans ^a^* | ND | ND | Cambodia | Sihanouk | FL | *Rattus tanezumi* | F | C0558 | |
| *L.interrogans* | H | 430/340/460 | Cambodia | Sihanouk | FL | *Rattus tanezumi* | M | C0572 | |
| *L.interrogans ^a^* | ND | ND | Cambodia | Sihanouk | F | *Rattus tanezumi* | M | C0592 | |
| *L.kirschneri ^a^* | ND | ND | Thailand | Loei | NFL | *Mus cervicolor* | F | R5974 | |
| *L.kirschneri* | ND | ND | Lao PDR | Luang Prabang | NFL | *Mus cookii* | M | L0064 | |
| *L.weilii* | ND | ND | Cambodia | Mondolkiri | HD | *Bandicota savilei* | F | C0458 | |

^a^ species identification after *rrs* nested-PCR

^b^ size length of PCR products of VNTR4, VNTR7, and VNTR10 (in basepairs)

**Table S2.** List of reference and clinical strains used in this study. We indicate information about species, serogroup, host, sampling and molecular results of the *secY* gene sequencing (alleles A to G) and MLVA typing. ND= non-determined.

| ***Leptospira* sp** | **Serogroup** | **Strain** | **Host** | **Country** | **Locality** | **Year** | ***secY*** | **MLVA** |
| --- | --- | --- | --- | --- | --- | --- | --- | --- |
| *L.interrogans* | Autumnalis | 1263 | Human | Thailand | Buriram | 2003 | ND | 450/750/740 |
| *L.interrogans* | Autumnalis | 86 * | Human | Thailand | Loei | 2001 | C | 480/650/750 |
| *L.interrogans* | Autumnalis | 146* | Human | Thailand | Buriram | 2001 | C | 480/650/750 |
| *L.interrogans* | Autumnalis | L 20 * | Human | Thailand | Udon Thani | 2002 | C | 480/650/750 |
| *L.interrogans* | Autumnalis | L 25 * | Human | Thailand | Udon Thani | 2002 | C | 480/650/750 |
| *L.interrogans* | Autumnalis | L 88 * | Human | Thailand | Udon Thani | 2002 | C | 480/650/750 |
| *L.interrogans* | Canicola | 136/2/2 # | *Rattus norvegicus* | Kuwait | Kuwait city | 1979 | E | 430/350/460 |
| *L.interrogans* | Canicola | TOA13A | *Rattus norvegicus* | Madagascar | Toamasina | 2008 | E | 430/350/460 |
| *L.interrogans* | Canicola | TOA25R | *Rattus norvegicus* | Madagascar | Toamasina | 2008 | E | 430/350/460 |
| *L.interrogans* | Javanica | 638 | Human | Thailand | Loei | 2002 | ND | 450/750/650 |
| *L.interrogans* | Pyrogenes | 95 | Human | Thailand | Loei | 2001 | D | 640/750/650 |
| *L.interrogans* | ND | 295 | Human | Thailand | Buriram | 2001 | ND | 480/650/750 |
| *L.interrogans* | ND | 11-1-167* | Human | Thailand | Nakhon Rachasima | 2011 | C | 480/650/750 |
| *L.interrogans* | ND | 11-1-206* | Human | Thailand | Nakhon Rachasima | 2011 | C | 480/650/750 |
| *L.interrogans* | ND | 11-BC-064* | Human | Thailand | Buriram | 2011 | C | 480/650/750 |
| *L.interrogans* | ND | NAN 028* | Human | Thailand | Nan | 2011 | C | 480/650/750 |
| *L.kirschneri* | ND | BC 68 | Human | Thailand | Buriram | 2009 | ND | 360/330/- |
| *L.weilii* | ND | NAN 033 | Human | Thailand | Nan | 2011 | ND | negative |

* clone ST34 (Thaipadungpanit et al. 2007)

# reference strain (serovar Kuwait)

**Table S3.** General Linear Model of rodent infection by *Leptospira* *borgpetersenii* with binomial distribution and logit link function (Log-Likelihood Type 1 Test). Selection of the best model using AIC criterion, with initial model with locality, habitat, species, sex and maturity as explicative variables.

| **Variables** | **Estimate**  **(SD)** | **p-values** | **Deviance** | **Degree freedom** | **AIC** |
| --- | --- | --- | --- | --- | --- |
| Sex: Male | 0.67 (0.39) | 0.089 |  |  |  |
| Habitat: flooded lands | 0.17 (0.55) | 0.759 |  |  |  |
| Habitat: non-flooded lands | -0.13 (0.51) | 0.799 |  |  |  |
| Habitat: Human dwellings | -2.38 (1.11) | 0.031* |  |  |  |
| Locality: Buriram | - | - |  |  |  |
| Locality: Champasak | -2.03 (1.06) | 0.055 |  |  |  |
| Locality: Luang Prabang | -1.89 (0.86) | 0.028* |  |  |  |
| Locality: Mondolkiri | -1.83 (0.73) | 0.012* |  |  |  |
| Locality: Nan | - | - |  |  |  |
| Locality: Sihanouk | -3.02 (1.03) | 0.003** |  |  |  |
| Intercept | -1.79 (0.54) | 0.001** |  |  |  |
|  |  |  | 290.8 | 837 | 233.6 |

* significant; ** very significant; *** highly significant

**Table S4.** General Linear Model of rodent infection by *Leptospira* *interrogens* with binomial distribution and logit link function (Log- Likelihood Type 1 Test). Selection of the best model using AIC criterion, with initial model with locality, habitat, species, sex and maturity as explicative variables.

| **Variables** | **Estimate**  **(SD)** | **p-values** | **Deviance** | **Degree freedom** | **AIC** |
| --- | --- | --- | --- | --- | --- |
| Sex: Male | 0.69 (0.45) | 0.126 |  |  |  |
| Habitat: flooded lands | -0.16 (0.69) | 0.810 |  |  |  |
| Habitat: non-flooded lands | -1.45 (0.71) | 0.041* |  |  |  |
| Habitat: Human dwellings | -1.81 (0.74) | 0.014* |  |  |  |
| Locality: Buriram | - | - |  |  |  |
| Locality: Champasak | - | - |  |  |  |
| Locality: Luang Prabang | 1.35 (0.89) | 0.133 |  |  |  |
| Locality: Mondolkiri | -0.38 (1.26) | 0.760 |  |  |  |
| Locality: Nan | 1.12 (1.04) | 0.280 |  |  |  |
| Locality: Sihanouk | 1.97 (0.83) | 0.017* |  |  |  |
| Intercept | -4.30 (0.89) | <0.001*** |  |  |  |
|  |  |  | 224.8 | 837 | 198.3 |

* significant; ** very significant; *** highly significant
